# Maresin conjugates in tissue regeneration-1 suppresses ferroptosis in septic acute kidney injury

**DOI:** 10.1186/s13578-021-00734-x

**Published:** 2021-12-27

**Authors:** Ji Xiao, Qian Yang, Ye’an Zhang, Haoran Xu, Yang Ye, Linchao Li, Yi Yang, Shengwei Jin

**Affiliations:** Department of Anesthesia and Critical Care, The Second Affiliated Hospital and Yuying Children’s Hospital of Wenzhou Medical University, Wenzhou, 325027 China

**Keywords:** Maresin conjugates in tissue regeneration 1, Ferroptosis, Sepsis, Acute kidney injury, Nuclear factor-erythroid-2-related factor 2

## Abstract

**Background:**

Ferroptosis is unique among different types of regulated cell death and closely related to organ injury. Whether ferroptosis occurs in sepsis-associated acute kidney injury (SA-AKI) is not clear. Nuclear factor-erythroid-2-related factor 2 (Nrf2) is crucial to the regulation of ferroptosis. We and others have shown that Maresin conjugates in tissue regeneration 1 (MCTR1) or other members of specialized pro-resolving mediators (SPMs) can actively regulate inflammation resolution and protect organs against injury in inflammatory diseases by activating the Nrf2 signaling. The aim of this study was to determine whether ferroptosis occurs in SA-AKI. Furthermore, we investigated the potential role and mechanism of MCTR1 in the regulation of ferroptosis in SA-AKI, which mainly focus on the Nrf2 signaling.

**Results:**

We demonstrated for the first time that ferroptosis is present in SA-AKI. Moreover, MCTR1 effectively suppressed ferroptosis in SA-AKI. Meanwhile, MCTR1 upregulated the expression of Nrf2 in the kidney of septic mice. Nrf2 inhibitor ML-385 reversed MCTR1-regulated ferroptosis and AKI, implying that Nrf2 is involved in the inhibitory effects of MCTR1 on ferroptosis in SA-AKI. Further, MCTR1 inhibited ferroptosis and elevated the expression of Nrf2 in LPS-induced HK-2 cells. However, Nrf2 siRNA offset the effect of MCTR1 on ferroptosis. Finally, we observed that MCTR1 ameliorates multi-organ injury and improves survival in animal models of sepsis.

**Conclusions:**

These data demonstrate that MCTR1 suppresses ferroptosis in SA-AKI through the Nrf2 signaling. Our study enriches the pathophysiological mechanism of SA-AKI and provides new therapeutic ideas and potential intervention targets for SA-AKI.

**Supplementary Information:**

The online version contains supplementary material available at 10.1186/s13578-021-00734-x.

## Introduction

Sepsis has long been considered the chief cause of acute kidney injury (AKI) in intensive care unit [1]. AKI from sepsis is also known as sepsis-associated acute kidney injury (SA-AKI). SA-AKI significantly prolongs the length of hospital stay and increases the in-hospital mortality in patients with severe sepsis. It is worth noting that some survivors are still at long-term risk of developing chronic kidney disease (CKD) or end-stage renal disease (ESRD) [2]. Sepsis makes SA-AKI a condition different from any other AKI phenotype. Currently, the paradigm for explaining SA-AKI seems to gradually change from vasoconstriction and hypoperfusion to vasodilation and hyperemia, from acute tubular necrosis (ATN) to tubular cell apoptosis, dysfunction or shedding [3–5]. Evidence of tubular cell death does exist in SA-AKI. Previous studies have shown that renal tubular cell necrosis, apoptosis, and autophagy occur in SA-AKI [6–11]. However, these known modes of cell death in the kidneys cannot fully explain the changes in SA-AKI.

Ferroptosis, a unique form of regulated cell death, was proposed by Stockwell et al. in 2012 [12]. It is characterized by a lethal accumulation of iron-dependent lipid hydroperoxides. The dying cells have no nuclear condensation, deoxyribonucleic acid (DNA) fragmentation, and caspase activation. Interestingly, the only distinctive morphological feature of ferroptotic cells is that the mitochondria are smaller and with membrane density increased [13]. Specific inhibitors of apoptosis, pyroptosis, necrosis, and autophagy could not reverse this process. However, iron chelators and antioxidants can inhibit this process. Mechanically, excessive iron accumulation, enhanced lipid peroxidation, and ineffective elimination of lipid peroxides are still the pathophysiological basis of ferroptosis [14]. Recently, studies have shown that ferroptosis is a primary contributor in models of organ injury, including degenerative brain disorders, ischemic injury to the heart, liver, intestine as well as other [15]. Up to now, it is not clear whether ferroptosis exists in SA-AKI.

Ferroptosis is tightly controlled by iron, amino acid, and lipid metabolism. Alterations in the metabolic homeostasis potentially affect the sensitivity of cells to ferroptosis [14]. The nuclear factor-erythroid-2-related factor 2 (Nrf2) belongs to the Cap “n” collar family of basic region leucine zipper transcription factors (CNC-bZip). Nrf2 controls the expression of critical proteins in iron metabolism, including ferritin (FTH) and ferroportin (FPN), and the expression of key enzymes in the detoxification/antioxidant responses, including NAD(P)H: quinone oxidoreductase 1 (NQO1) and sestrin 2. Additionally, genes encoding proteins contribute to glutathione synthesis and metabolism, including solute carrier family 7 member 11 (SLC7A11) and glutathione synthase (GSS) are Nrf2 target genes [16]. Thus, Nrf2 is potentially involved in the regulation of ferroptosis. Indeed, previous studies have shown that Nrf2 was involved in the tolerance of tumor cells to ferroptosis inducer [17]. Apart from cancer, Nrf2 also mediated the resistance of normal cells and tissues to multiple etiological factors induced ferroptosis in a variety of diseases [18]. Collectively, the Nrf2 is important to ferroptosis, and pharmacological activation of Nrf2 may be a potential treatment for diseases to which preventing ferroptosis is more advantageous.

The specialized pro-resolving mediators (SPMs) family includes resolvins, lipoxins, protectins, and maresins [19]. Their ability to actively regulate inflammation resolution and organ protection has been widely studied in our and other laboratories [20–24]. Previous studies have shown that the Nrf2 signaling is involved in the pro-resolution and organ protection of Maresin 1 [25, 26]. Other members of SPMs such as lipoxin A4 also play a protective role in many inflammatory diseases by activating the Nrf2 signaling [27–29]. Maresin conjugates in tissue regeneration 1 (MCTR1), a new member of SPMs, is the product of Maresin 1 transformed by glutathione S-transferase mu4 or leukotriene C4 synthase [30]. We previously demonstrated that MCTR1 could protect against septic cardiomyopathy at least partly through sirtuin-1/Nrf2 pathway mediated mitochondrial biogenesis and function [31]. These evidences suggest that Nrf2 may be a potential target to mediate the biological function of MCTR1. Thus, this study was designed to determine whether ferroptosis occurs in SA-AKI. Furthermore, we investigated the potential role and mechanism of MCTR1 in the regulation of ferroptosis in SA-AKI, which mainly focus on the Nrf2 pathway.

## Results

### Ferroptosis is present in CLP-induced AKI

**Fig. 1:**
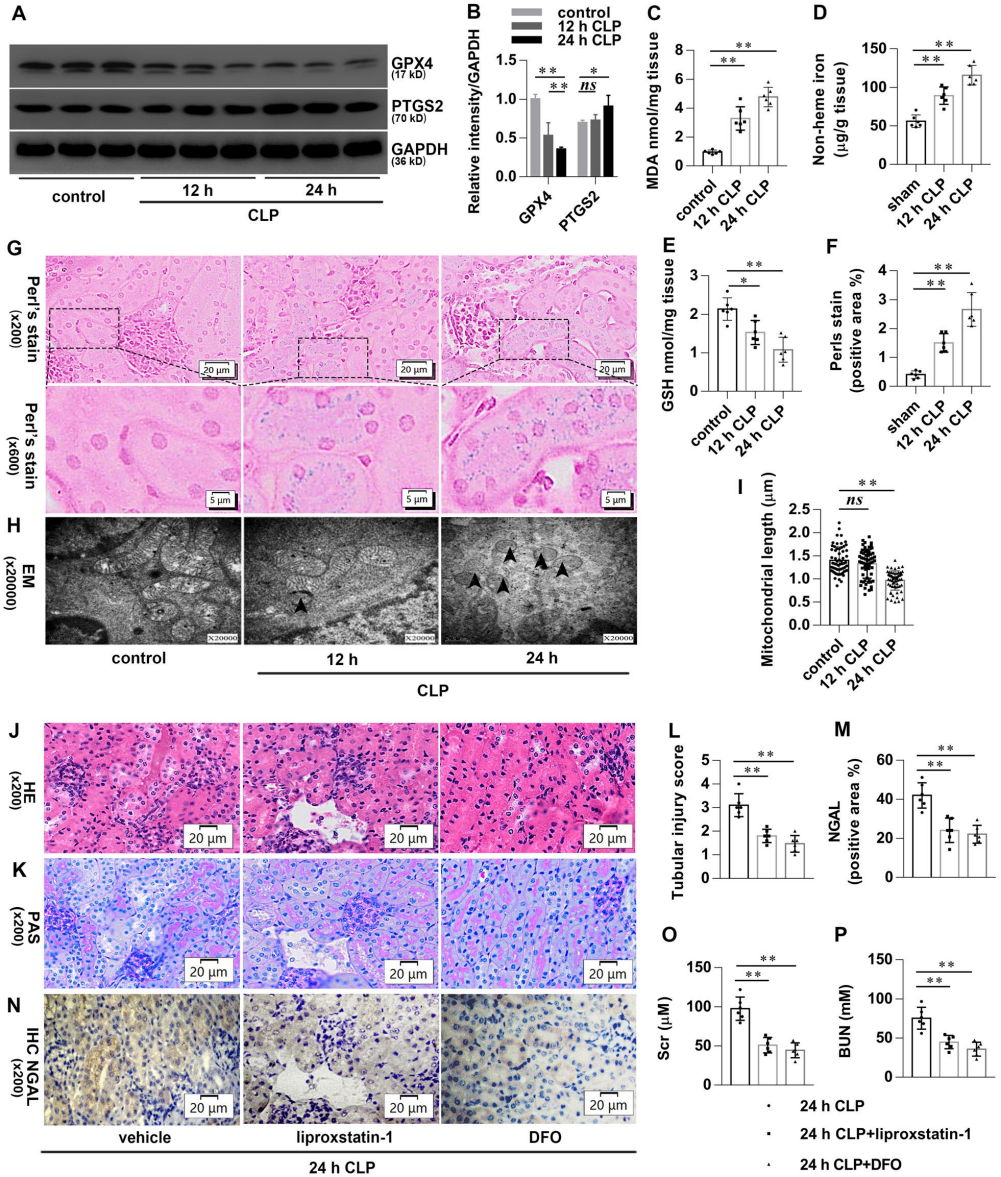
Ferroptosis is present in CLP-induced AKI. Mice were subjected to CLP, then the kidneys were collected at the indicated time. **A**, **B** Shown are representative western blotting and quantification of GPX4 and PTGS2. **C–E** Quantitative analyses of MDA, GSH, and non-heme iron. **F** Quantitative analyses of Perl’s stain. **G** Representative Perl’s stain images. **H** Representative transmission electron microscopy (TEM) images. The black arrow indicates ferroptosis-like mitochondria. **I** Quantitative analyses of mitochondrial length. Mice were pretreated with liproxstatin-1 (10 mg/kg, ip) or DFO (20 mg/kg, ip) 0.5 h before being subjected to CLP. All kidney samples were collected at 24 h after CLP. **J–K** Shown are representative Hematoxylin-eosin (HE) stains and Periodic Acid-Schiff (PAS) stain. **L** Histological analyses of renal tubular injury. **M** Quantitative analyses of IHC stain of NGAL. **N** Representative immunohistochemistry (IHC) images for NGAL. **O**, **P** Quantitative analyses of Scr and BUN. n = 6 mice/group, mean ± SD were presented. *P < 0.05, **P < 0.01 and *ns*: *P > 0.05*. ip: intraperitoneal

We first examined the effect of caecal ligation and puncture (CLP) on acute kidney injury (AKI). As expected, time course experiments revealed that renal pathology (Additional file 1:  Fig. S1A–E) and function (Additional file 1: Fig. S1F, G) deteriorated after CLP in a time-dependent manner. We then observed ferroptotic morphological and biochemical changes in CLP-induced AKI. Glutathione peroxidase 4 (GPX4) and prostaglandin-endoperoxide synthase 2 (PTGS2) are well-known ferroptotic markers. As shown in Fig. 1A, B, the expression of GPX4 was significantly reduced in the kidneys after CLP. Meanwhile, the expression of PTGS2 exhibited a relatively lower increase at 12 h and obliviously at 24 h. Besides, end product of lipid peroxidation malondialdehyde (MDA) and non-heme iron in the renal tissues were also significantly increased, accompanied by glutathione (GSH) content significantly decreased after CLP (Fig. 1C–E). Perls’ Prussian Blue stain shown that heme-iron in the renal tissues was significantly increased after CLP (Fig. 1F, G). Transmission electron microscopy shown that the length of mitochondria in renal tubular epithelial cells (TECs) of the CLP group shortened at 12 h and significantly shorter at 24 h. Typical ferroptotic mitochondria with high bilayer density were found at 24 h after CLP (Fig. 1H, I). We finally determine the effect of specific inhibitor of ferroptosis on CLP-induced AKI. As shown in Fig. 1J–L, TECs vacuolization, tubules dilated, and brush border injury occurred commonly in CLP mice, accompanied by scattered tubules cast and exfoliated cells. However, these pathological damages were reduced significantly in CLP mice when pretreated with liproxstatin-1 or deferoxamine (DFO). Neutrophil gelatinase-associated lipocalin (NGAL) is a sensitive biomarker for early diagnosis of AKI. Immunohistochemical staining showed that the expression of NGAL in renal tubules was significantly increased at 24 h after CLP, which was significantly reversed by liproxstatin-1 or DFO pretreatment (Fig. 1M, N). Similarly, the elevated serum creatinine (Scr) and blood urea nitrogen (BUN) in CLP mice were significantly improved by liproxstatin-1 or DFO pretreatment (Fig. 1O, P). These data suggest that ferroptosis is present in CLP-induced AKI.

### MCTR1 suppresses ferroptosis in CLP-induced AKI

**Fig. 2:**
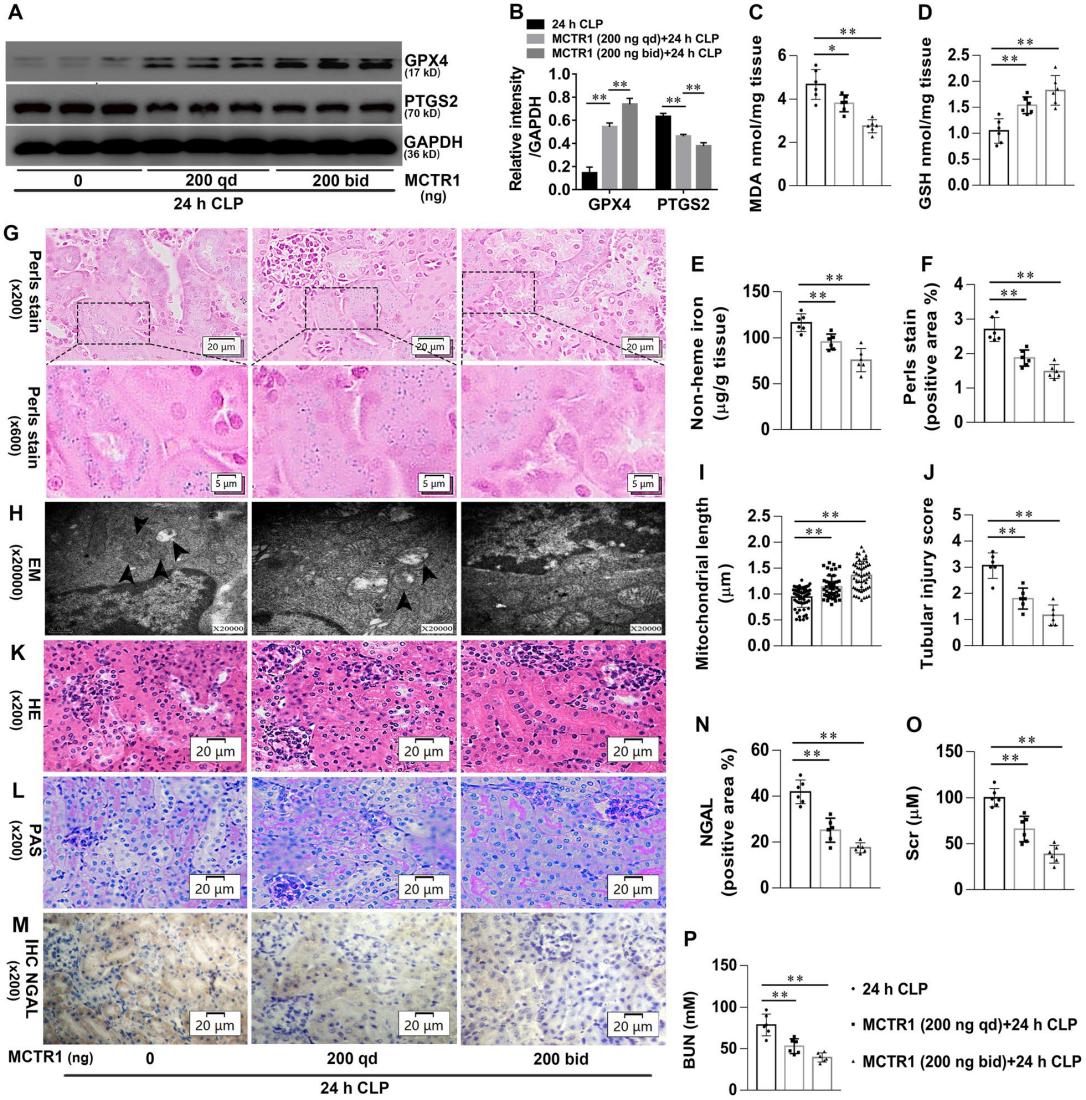
Effects of MCTR1 on ferroptosis in CLP-induced AKI. Mice were given MCTR1 once, twice, or not during the 24 h experiment. The once-daily administration mode (qd): Mice were given MCTR1 (200 ng/mice, iv) 0.5 h before being subjected to CLP. The twice-daily administration mode (bid): Mice were given MCTR1 (200 ng/mice, iv) 0.5 h before being subjected to CLP, and then an additional MCTR1 (200 ng/mice, iv) was given 12 h after CLP. All kidney samples were collected at 24 h after CLP. **A**, **B** Shown are representative western blotting and quantification of GPX4 and PTGS2. **C–E** Quantitative analyses of MDA, GSH, and non-heme iron. **F** Quantitative analyses of Perl’s stain. **G** Representative Perl’s stain images. **H** Representative TEM images. The black arrow indicates ferroptosis-like mitochondria. **I** Quantitative analyses of mitochondrial length. **J** Histological analyses of renal tubular injury. **K**, **L** Shown are representative HE stains and PAS stain. **M** Representative IHC images for NGAL. **N** Quantitative analyses of IHC stain of NGAL. **O**, **P** Quantitative analyses of serum Scr and BUN. n = 6 mice/group, mean ± SD were presented. **P < 0.01. iv: intravenous

We next to verify whether MCTR1 regulates ferroptosis in CLP-induced AKI. As shown in Fig. 2A, B, MCTR1 significantly increased the expression of GPX4 and decreased the expression of PTGS2 in the kidneys after CLP in a dose-dependent manner. MCTR1 also significantly decreased MDA, non-heme iron, and heme-iron, accompanying with GSH content increased significantly (Fig. 2C–G). Moreover, the length of mitochondria in TECs of the MCTR1 groups was significantly longer when compared with the CLP group (Fig. 2H, I). These data suggest that MCTR1 suppresses ferroptosis in CLP-induced AKI. We further determined the effect of MCTR1 on CLP-induced AKI. As shown in Fig. 2J–L, TECs vacuolization, tubules dilated, brush border injury, scattered tubules cast, and exfoliated cells were decreased significantly in the MCTR1 groups when compared with the CLP group. Immunohistochemical staining showed that the expression of NGAL in renal tubules was remarkably decreased in the MCTR1 group compared with the CLP group (Fig. 2M, N). Similarly, the renal function also markedly improved by showing a decrease of Scr and BUN in the MCTR1 group (Fig. 2O, P). These data suggest that MCTR1 suppresses ferroptosis and exhibits a protective effect on CLP-induced AKI.

### Nrf2 mediates the inhibitory effects of MCTR1 on ferroptosis in CLP-induced AKI

**Fig. 3:**
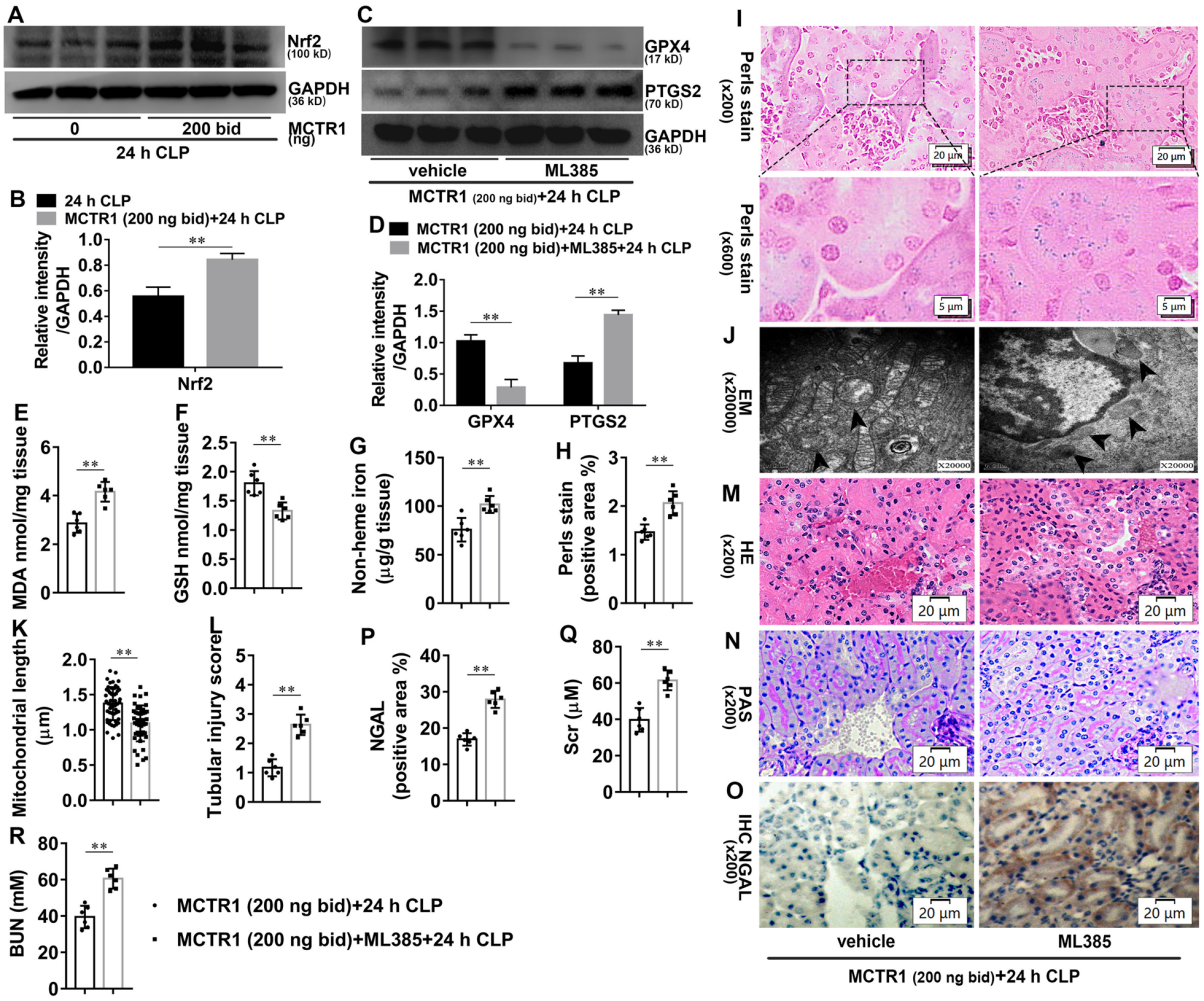
Effects of Nrf2 on MCTR1-regulated ferroptosis in CLP-induced AKI. Mice were given ML385 (30 mg/kg, ip, qd) for 7 d. On day 7, ML385 injection with or without a twice-daily administration mode of MCTR1 as described before in the following experiment. All kidney samples were collected at 24 h after CLP. **A–D** Shown are representative western blotting and quantification of Nrf2, GPX4, and PTGS2. **E**–**G** Quantitative analyses of MDA, GSH, and non-heme iron. **H** Quantitative analyses of Perl’s stain. **I** Representative Perl’s stain images. **J** Representative TEM images. The black arrow indicates ferroptosis-like mitochondria. **K** Quantitative analyses of mitochondrial length. **L** Histological analyses of renal tubular injury. **M**, **N** Representative HE stains and PAS stain. **O** Representative IHC images for NGAL. **P** Quantitative analyses of IHC stain of NGAL. **Q**, **R** Quantitative analyses of serum Scr and BUN. n = 6 mice/group, mean ± SD were presented. **P < 0.01

To investigate the effect of the Nrf2 on MCTR1-regulated ferroptosis in CLP-induced AKI, Nrf2 proteins were detected by western blotting. As shown in Fig. 3A, B, twice-daily administration with MCTR1 significantly increased the expression of Nrf2 in the kidneys of mice at 24 h after CLP. We then investigated the role of Nrf2 on MCTR1 regulated-ferroptosis in CLP-induced AKI. As shown in Fig. 3C, D, Nrf2 inhibitor ML-385 significantly decreased the expression of GPX4 and increased the expression of PTGS2 in ML-385 plus MCTR1 group compared with the MCTR1 group at 24 h after CLP. Meanwhile, ML-385 significantly increased MDA, non-heme iron, and heme-iron, accompanying with GSH content decreased (Fig. 3E–I). Besides, the length of mitochondria in TECs of ML-385 plus MCTR1 group was significantly shorter when compared with the MCTR1 group at 24 h after CLP (Fig. 3J, K). Similarly, renal pathology (Fig. 3L–P) and function (Fig. 3Q, R) deteriorated obviously in ML-385 plus MCTR1 group compared with the MCTR1 group at 24 h after CLP. These data suggest that Nrf2 is involved in MCTR1-regulated ferroptosis in CLP-induced AKI.

### MCTR1 inhibits LPS-induced ferroptosis in vitro

**Fig. 4:**
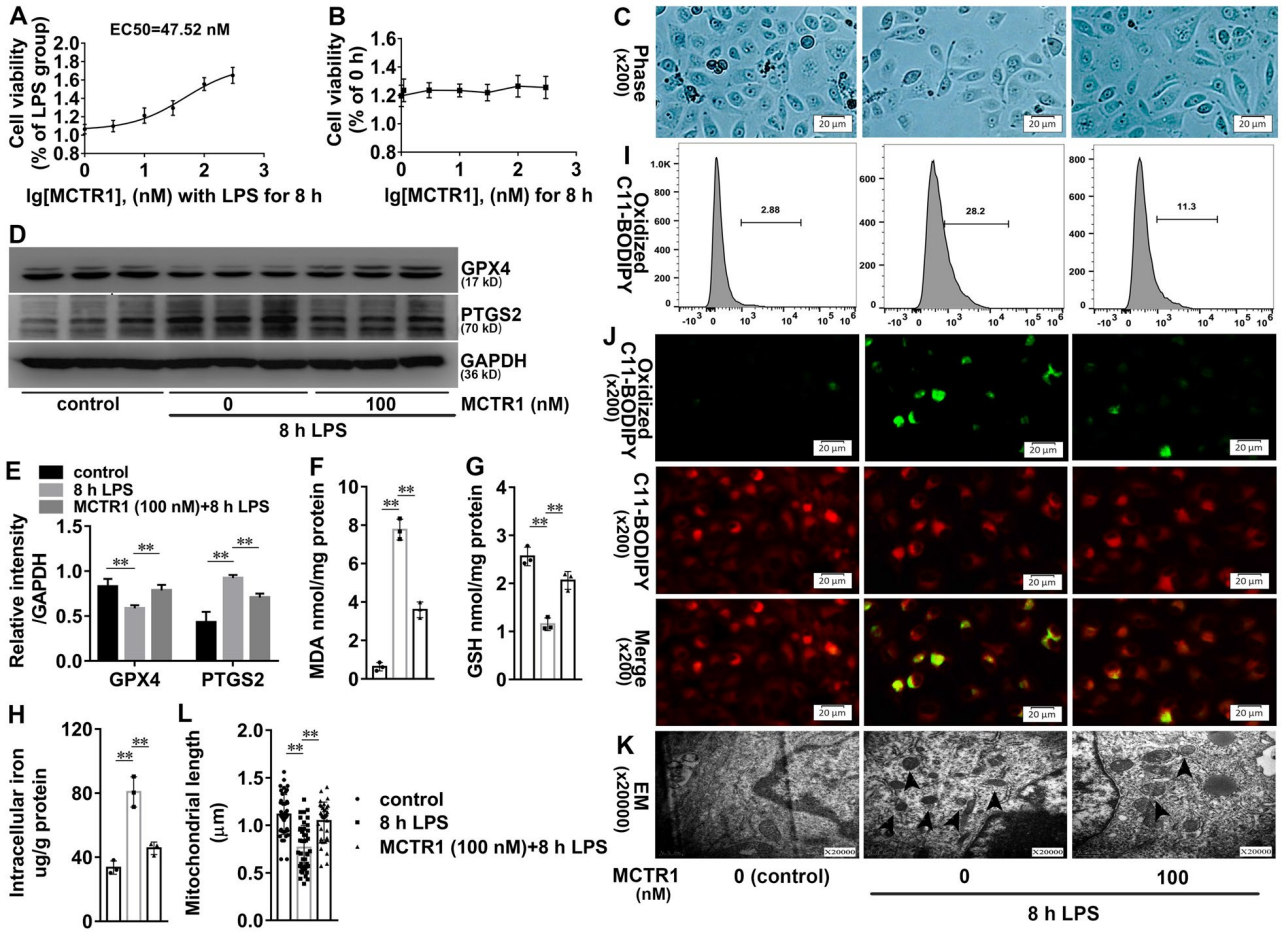
Effects of MCTR1 on LPS-induced lipid peroxidation and ferroptosis in vitro. **A** Viability curves for HK-2 cells treated with different concentrations (0, 1, 3, 10, 30, 100, and 300 nM) of MCTR1 and LPS (1 ug/ml) for 8 h. **B** Viability curves for HK-2 cells treated with different concentrations (0, 1, 3, 10, 30, 100, and 300 nM) of MCTR1 for 0 and 8 h. **C** HK-2 cells treated with MCTR1 (100 nM) and LPS (1 ug/ml) for 8 h, and the visualization of cell viability were evaluated by phase-contrast microscopy. **D**, **E** Representative western blotting and quantification of GPX4 and PTGS2 protein. **F**–**H** Quantitative analyses of MDA, GSH and, non-heme iron. **I** Quantitative analyses of oxidized C11-BODIPY 581/591 probe by flow cytometry. **J** Representative images of C11-BODIPY 581/591 fluorescent ratio-probe. **K** Representative TEM images. The black arrow indicates ferroptosis-like mitochondria. **L** Quantitative analyses of mitochondrial length. n = 3, mean ± SD were presented. **P < 0.01

Lipopolysaccharide (LPS) is the leading cause of inflammatory reaction and organ damage in sepsis. To further validate the effect of MCTR1 on ferroptosis in vitro, LPS was used first. As shown in Additional file 2:  Fig. S2A, B, LPS inhibited cell viability in a time and dose-dependent manner. Liproxstatin-1 or DFO significantly elevated cell viability after LPS treatment, implying that ferroptosis is present in LPS-induced cell death (Additional file 2: Fig. S2 C, D). We then explored the effect of MCTR1 on LPS-induced cell viability. As shown in Fig. 4A, MCTR1 ameliorated LPS-induced decline of cell viability, and the concentration for 50% of maximal effect (EC50) is about 47.52 nM. Different concentrations of MCTR1 did not affect the cell proliferation at the indicated time, suggesting that MCTR1 may inhibit LPS-induced cell death (Fig. 4B). 100 nm MCTR1 significantly inhibited LPS-induced cell death (Fig. 4C). We next verify whether MCTR1 regulates LPS-induced ferroptosis in HK-2 cells. As shown in Fig. 4D, E, LPS inhibited the expression of GPX4 and induced the expression of PTGS2. However, MCTR1 reversed LPS-induced protein expression of GPX4 and PTGS2. LPS also significantly increased MDA, non-heme iron, and heme-iron, accompanying with GSH content decreased. These biochemical changes induced by LPS were also reversed by MCTR1 (Fig. 4F–H). In parallel, lipid reactive oxygen species (ROS) was observed by flow cytometry and fluorescence microscope. As shown in Fig. 4I, J, the production of oxidized C11 BODIPY 581/591 in the LPS group was increased compared with the control group. However, MCTR1 inhibited LPS-induced oxidized C11 BODIPY 581/591 production. Additionally, ferroptotic mitochondria were found in the LPS group. The length of mitochondria in the LPS plus MCTR1 group was significantly longer when compared with the LPS group (Fig. 4K, L). Directly, MCTR1 significantly inhibited erastin-induced ferroptosis in HK-2 cells (Additional file 3: Fig. S3A, B). These data suggest that LPS induces ferroptosis and MCTR1 inhibits ferroptosis in vitro.

### Nrf2 is required for the effects of MCTR1 on ferroptosis in vitro

**Fig. 5:**
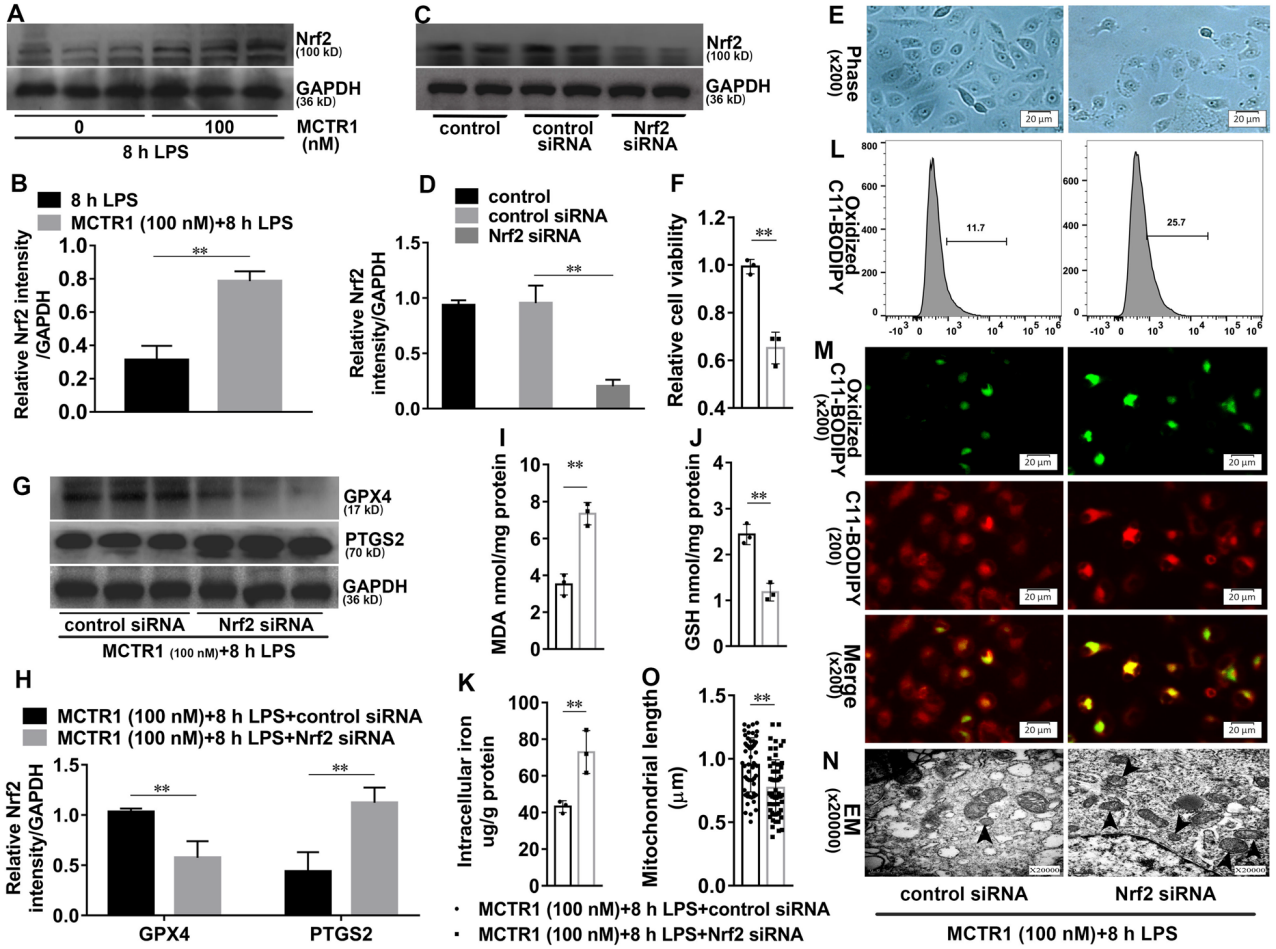
Nrf2 is involved in the inhibitory effects of MCTR1 on LPS-induced ferroptosis. **A**, **B** HK-2 cells were treated with MCTR1 (100 nM) and LPS (1 ug/ml) for 8 h. Shown are representative western blotting and quantification of Nrf2. HK-2 cells were transfected with 30 nM Nrf2 siRNA for 48 h and then treated with MCTR1 (100 nM) and LPS (1 ug/ml) for 8 h. **C**, **D** Representative western blotting and quantification of Nrf2. **E** Visualization of cell viability were evaluated by phase-contrast microscopy. **F** Fold change of cell viability. **G**, **H** Representative western blotting and quantification of GPX4 and PTGS2. **I**–**K** Quantitative analyses of MDA, GSH, and non-heme iron. **L** Quantitative analyses of oxidized C11-BODIPY 581/591 probe by flow cytometry. **M** Representative images of C11-BODIPY 581/591 fluorescent ratio-probe. **N** Representative TEM images. The black arrow indicates ferroptosis-like mitochondria. **O** Quantitative analyses of mitochondrial length. n = 3, mean ± SD were presented. **P < 0.01

We next investigate the effects of Nrf2 on MCTR1 regulated-ferroptosis. As shown in Fig. 5A, B, MCTR1 significantly increased the protein expression of Nrf2 in LPS-induced HK-2 cells. To further investigate the role of Nrf2 in MCTR1 regulated-ferroptosis in vitro, Nrf2 siRNA was used. As shown in Fig. 5C, D, Nrf2 siRNA significantly decreased the protein expression of Nrf2. Moreover, Nrf2 siRNA significantly reduced the cell viability with increased cell death in LPS with MCTR1 treated group (Fig. 5E, F). Nrf2 siRNA also significantly decreased the expression of GPX4 and increased the expression of PTGS2 in LPS with MCTR1 treated group (Fig. 5G, H). Meanwhile, Nrf2 siRNA treatment significantly increased MDA, non-heme iron, and oxidized C11 BODIPY 581/591 production, accompanying with GSH content significantly decreased (Fig. 5I–M). Additionally, the length of mitochondria in the MCTR (100 nM) + 8 h LPS + Nrf2 siRNA group was significantly shorter when compared with the MCTR (100 nM) +8 h LPS + control siRNA (Fig. 5N, O). Similarly, Nrf2 siRNA also offset the role of MCTR1 in erastin-induced ferroptosis in vitro (Additional file 3: Fig. S3C, D). These results demonstrate that Nrf2 is involved in the inhibitory effects of MCTR1 on ferroptosis in vitro.

### MCTR1 ameliorates multiple organ injury and improves survival in septic animal models

**Fig. 6:**
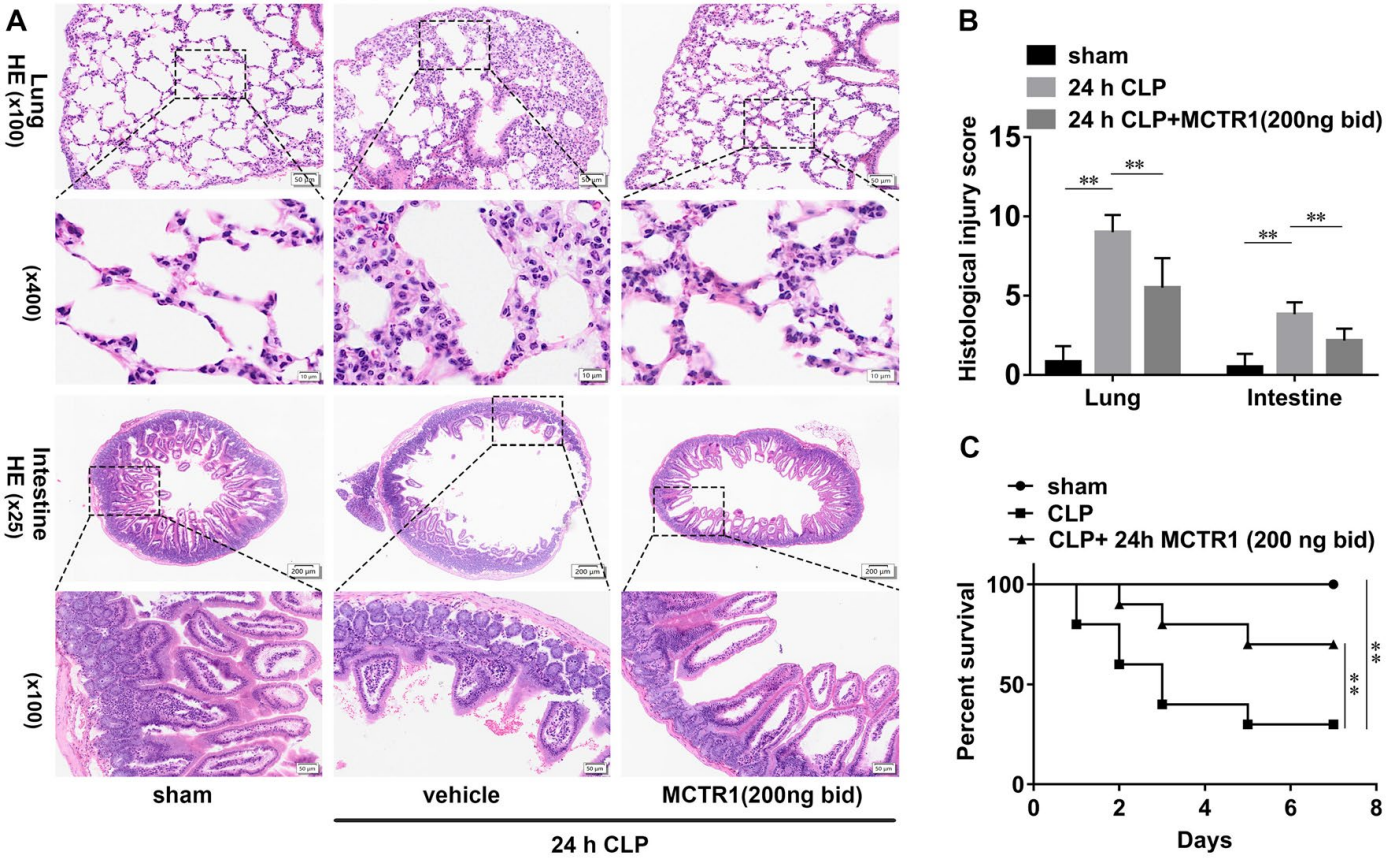
Effects of MCTR1 on ALI, intestinal mucosal damage, and survival in CLP-induced sepsis. **A** Mice were given MCTR1 (200 ng/mice, iv) 0.5 h before being subjected to CLP, and then an additional MCTR1 (200 ng/mice, iv) was given 12 h after CLP. All samples were collected 24 h after CLP. Representative HE stains images of lung and intestinal sections (n = 6 mice/group). **B** Histological analyses of CLP-induced ALI and AII. **C** Mice were given MCTR1 (200 ng/mice, iv) 0.5 h before being subjected to CLP, and then an additional MCTR1 (200 ng/mice, iv) was given 12 h after CLP. The survival rate (n = 10 mice/group) was recorded for 7 consecutive days. Mean ± SD were presented. **P < 0.01

Lung and intestine are most vulnerable to sepsis, and multiple organ injury is associated with increased mortality in patients with sepsis. To analyzed the overall effect of MCTR1 on sepsis, we then observed MCTR1 on CLP-induced lung injury, intestinal injury, and mortality. As shown in Fig. 6A–C, the CLP group displayed severe lung injury and intestinal injury compared with the sham group. Administration with MCTR1 obliviously improved the pathological changes caused by sepsis. Consistent with the pathological changes, mice subjected to CLP had a poor 7-day survival rate compared with the sham group. Administration with MCTR1 got a better effect on protecting mice against death (Fig. 6D). These data suggest that MCTR1 ameliorates multiple injuries and improves survival in sepsis.

## Discussion

Based on a comprehensive ferroptosis-related assessment, we demonstrated for the first time that ferroptosis is present in SA-AKI. We also observed that MCTR1 effectively suppressed ferroptosis in SA-AKI. Meanwhile, MCTR1 upregulated the expression of Nrf2 in the kidney of septic mice. Nrf2 inhibitor ML-385 reversed MCTR1-regulated ferroptosis and AKI, implying that Nrf2 is involved in the inhibitory effects of MCTR1 on ferroptosis in SA-AKI. To further clarify whether MCTR1 regulates ferroptosis, we verified it in vitro. Similarly, MCTR1 inhibited ferroptosis and elevated the expression of Nrf2 in LPS-induced HK-2 cells. However, Nrf2 siRNA offset the effect of MCTR1 on ferroptosis. Finally, we observed that MCTR1 ameliorates multi-organ injury and improves survival in animal models of sepsis. These data demonstrate that ferroptosis occurs in SA-AKI and MCTR1 effectively alleviates ferroptosis through the Nrf2 signaling in SA-AKI.

In the past, TECs death or ATN was thought to be responsible for SA-AKI [32, 33]. Unfortunately, extensive TECs death has not been described in the kidneys of SA-AKI patients and large animals, which leading investigators to postulate that cell death is not the basis of SA-AKI [11, 34–36]. In this study, our data from the CLP-induced sepsis model has firstly demonstrated that ferroptosis-related changes occur in the kidney. Moreover, ferroptosis inhibitor liproxstatin-1 and DFO markedly ameliorate CLP-induced AKI. Thus, we proposed that ferroptosis exists in SA-AKI. Our findings, therefore, are seen to be different from the previous observations. Several reasons may lead to this discrepancy. Firstly, most previous studies are mainly based on morphological observation under the optical microscope. However, cells dying by ferroptosis or before the point of ferroptosis exhibit few morphological changes under optical microscopy, only with characteristic damaged mitochondria by electron microscopy [12, 13, 37]. Secondly, the lack of reagents for studying ferroptosis such as GPX4 antibodies in large animal tissues further limits the researchers to discover this type of cell death in SA-AKI. Finally, the confounding factors derived from SA-AKI models, fluid resuscitation, vasopressor drugs, and others also produced bias among large animals, small animals, and clinical experiments.

Indeed, kidneys are sensitive to ferroptosis. Inducible GPX4 knockout in the kidney arises massive ferroptosis of renal tubular epithelia, which results in death due to acute renal failure [38]. In line with our study, many previous studies have confirmed that ferroptosis is directly involved in AKI induced by IR, cisplatin, folic acid, rhabdomyolysis, and others [39–43]. Taken together, evidence from our and others implies that ferroptosis may be a central pathophysiological process and potential therapeutic target for AKI, including SA-AKI.

Iron is required for the initiation and execution of ferroptosis. Iron overload makes the cells sensitive to ferroptosis. During sepsis, inflammation and hemolysis are inevitably elevated. Inflammatory mediators, particularly IL-6, can sequester iron in cells by regulating the hepcidin pathway [44]. The infiltration of inflammatory cells, especially macrophages, can result in heme-iron deposits [45]. Heme is involved in the pathogenesis of severe sepsis. Heme tissue deposition and “heme sensitization” cause high oxidative stress and damage to cells [46]. Besides, the kidney is one of the primary organs for hemoglobin clearance. The reabsorption of filtered hemoglobin in the proximal tubule has resulted in additional oxidative damage [47]. Therefore, it is not surprising that we found an increase in iron content both in SA-AKI animal and cell models.

Given the essential role of MCTR1 in inflammation, we then investigated the effects of MCTR1 on ferroptosis in SA-AKI. We found that MCTR1 inhibited ferroptosis in CLP-induced AKI. Meanwhile, MCTR1 obliviously mitigated CLP-induced AKI. These data suggest that MCTR1 may potentially suppress ferroptosis in SA-AKI. Also, CLP-induced lung and intestinal injury pathology and mortality were obliviously improved by MCTR1 treatment. Collectively, MCTR1 exhibited a protective effect on multiple organ injury in CLP-induced sepsis. Whether other members of SPMs have similar functions to MCTR1 in sepsis needs to been further investigated.

It should be noted that iron-catalyzed lipid peroxidation is a primary driver of ferroptosis. Given that Nrf2 is a principal regulator of antioxidant response that suppresses ferroptosis. We further investigated the role of Nrf2 in MCTR1 regulated-ferroptosis in SA-AKI. We found that MCTR1 significantly increased the expression of Nrf2 in CLP-induced AKI. Nrf2 inhibitor reversed MCTR1 regulated ferroptosis in AKI in CLP-induced sepsis. These data suggest that Nrf2 mediates the protective effect of MCTR1 on AKI in CLP-induced sepsis. Similar to other SPMs, Nrf2 signaling plays a vital role in the biological function of MCTR1. All the same, further studies are needed to determine the mechanism by which MCTR1 affects Nrf2.

Recent studies have shown that ferroptosis was associated with sepsis-related liver and lung injury [48, 49]. However, the etiology of ferroptosis in sepsis has not been fully elucidated. LPS is a central pathogen-associated molecular pattern (PAMP) for sepsis with Gram-negative bacteria. Its receptor, Toll-like receptor 4 (TLR4), was expressed in the renal tubular epithelial cell (TECs) membrane [50]. When combined with LPS, the downstream cascade signal is triggered, resulting in increased oxidative stress and cell death. Here, we showed that ferroptosis was present in LPS-induced HK-2 cells. MCTR1 also inhibited LPS-induced ferroptosis through the Nrf2. Besides, a recent study has demonstrated that ferroptosis is involved in LPS-induced cardiomyocyte death and cardiomyopathy [51]. Together, ferroptosis is involved in sepsis-related organ injury, and LPS may induce ferroptosis. It is noteworthy that LPS also triggers apoptosis, autophagy, pyroptosis, necroptosis as well as other modes of regulated cell death [52–54]. Sepsis is a complex syndrome. Further researches are needed to clarify the principal mode and degree of cell death in different organs.

Dysregulated inflammatory responses and organ dysfunction are the core events of sepsis. In the past, researches focused on the regulation of inflammation initiation and persistence. The developed drugs based on this concept have been clinically proved ineffective during sepsis [55]. Recently, emphasis has tried to shift to inflammation resolution. It is now clear that SPMs actively promote resolution and tissue repair without compromising host defense [24]. In the study, we demonstrated that MCTR1 ameliorates multi-organ injury and improves survival in animal models of sepsis. Although MCTR1 appears to be an attractive new therapeutic target for sepsis, it has several inherent limitations that need to be addressed before being used clinically. For example, the administration time of MCTR1 may be critical in sepsis. The strict spatiotemporal regulation of MCTR1 may be hard to reproduce in humans, where determining the onset of an inflammatory attack can be challenging. In addition, the differences of pharmacokinetics of MCTR1, bacterial types and immune functions between small animals and humans should also be considered.

In conclusion, this study is the first to demonstrate that MCTR1 effectively alleviates SA-AKI, at least in part, by inhibiting ferroptosis. Mechanistically, MCTR1 activates the Nrf2, which may contribute to the inhibition of ferroptosis. This study enriched the pathophysiological mechanism of SA-AKI and provided new therapeutic ideas and potential intervention targets for SA-AKI.

## Materials and methods

### Reagents

MCTR1 was purchased from Cayman Chemical (Ann Arbor, MI, USA). LPS (L2637, Escherichia coli 055: B5), ML385, DFO, erastin, and liproxstain-1 were purchased from Sigma (St.Louis, MO, USA). Antibodies against GPX4, NRF/NFE2L2, and NGAL/LCN2 were purchased from BOSTER (Wuhan, China). Antibodies against PTGS2 and glyceraldehyde-3-phosphate dehydrogenase (GAPDH) were purchased from Abcam (Cambridge, UK). Antibodies against horseradish peroxidase (HRP)-conjugated goat anti-rabbit IgG secondary antibody were obtained from Santa Cruz Biotechnology (Santa Cruz, CA, USA).

### Animals and Caecal ligation and puncture (CLP) model

Male C57BL/6 mice aged 8–12 weeks, weighing about 25 g, were purchased from Shanghai Experimental Animal Center of China. Mice were housed in specific pathogen-free rooms with temperature (20–26 °C) and humidity (50–60%). A 12-h light–dark cycle (8:00 to 20:00 light) per day was performed. During the experiment, mice had free access to food and water. All the experimental protocols were strictly by the guidelines for the care and use of laboratory animals issued by the National Institutes of Health and approved by the ethics committee of experimental animals in the Second Affiliated Hospital of Wenzhou Medical University.

A mid-grade form of sepsis was induced by CLP as previously described [56]. Briefly, the mice were anesthetized with isoflurane. The abdomen area was disinfected after shaving. A 1 cm midline incision was made on the lower abdomen skin to expose the cecum. A 5-0 silk thread was used to ligate the midpoint between the distal pole and the bottom of the cecum. A 21 g needle was used to penetrate the cecum twice from the mesenteric toward the antimesenteric direction. After the wound was sutured, 0.25% ropivacaine was injected subcutaneously for postoperative analgesia. The mice were immediately resuscitated by injecting prewarmed normal saline 1 ml subcutaneously.

### Cell culture

HK-2 cells (Cell Resource Center, IBMS, CAMS&PUMC, Beijing, China) were cultured in Dulbecco’s Modified Eagle Medium/Ham’s F-12 (DMEM/F12) containing 1% penicillin-streptomycin solution and 10% fetal bovine serum. The cells were cultured in a humidified constant temperature incubator at 37 °C with 5% carbon dioxide. When the cell growth and fusion reached 70–80% (about 2–3 days), the passage was carried out. The third to fifth-generation HK-2 cells were used in the experiment.

### Cell viability

Cell viability was checked using Cell Counting Kit-8 (CCK-8, CA1210, Solarbio, Beijing, China) according to the manufacturer’s instructions.

### siRNA transfection

Cells were transfected with control siRNA (sc-37007, Santa Cruz, CA, USA) or siRNA against Nrf2 using the Nrf2 siRNA (sc-37,030, Santa Cruz, CA, USA) transfection reagent according to the manufacturer’s protocol. The siRNA-induced Nrf2 gene silencing was confirmed using western blotting. Assay the cells using the appropriate protocol 48 h after transfection.

### Lipid ROS assay

Cellular lipid ROS accumulation was measured using C11 BODIPY 581/591 (Invitrogen, Carlsbad, CA, USA) as a molecular probe. Briefly, the HK-2 cells were treated with LPS or LPS plus MCTR1 for 8 h. The original medium was replaced by a 1 ml medium containing 5 μm C11 BODIPY 581/591 dye, and the cells were cultured for an additional 30 min. At the end time point, cells were washed with PBS three times, then added with 2 ml medium, and finally observed under a fluorescence microscope. For quantitative analyses of lipid ROS, flow cytometry was used. Cell culture was the same as before. The cells were harvested, washed twice with PBS, and then suspended in 500 µl PBS. Oxidized C11-BODIPY 581/591 probe was detected by an InvitrogenTM AttuneTM NxT Flow Cytometer (Invitrogen, Carlsbad, CA, USA) and analyzed by FlowJo software.

### Western blotting

Renal tissue and HK-2 cell proteins were extracted. Protein concentrations were then quantified using a BCA protein assay kit (Rockford, IL, USA). 30 µg protein sample per hole was loaded onto 10% or 12% sodium dodecyl sulfate polyacrylamide gel electrophoresis (SDS-PAGE). The proteins were transferred to the Poly(vinylidene fluoride) (PVDF) membranes after electrophoretic separation. After the PVDF membranes were blocked with 5% skimmed milk for 2 h, the primary antibodies were incubated overnight at 4 °C. After the second antibody was incubated, the protein bands were finally visualized by ECL plus chemiluminescence substrate method. Relative quantification of proteins was performed with ImageJ software v1.8.0.

### Histological analysis

Kidney, lung, and intestine specimens were separated and fixed in Bouin’s fixative, then dehydrated, embedded in paraffin. The fixed samples were then sectioned (thickness, 4 μm) for hematoxylin and eosin (H&E), Periodic Acid-Schiff stain (PAS), Perls’ Prussian Blue, and immunohistochemistry (IHC) staining. The images were obtained and saved as digital images using Olympus VS200 Virtual Slide System (VS200, Olympus, Tokyo, Japan). Tubular injury was scored using a 5-point scale based on the percentage of brush border loss, tubular dilatation, cast deposition, and necrosis [57]. The scoring criteria are as follows: 0 point, no involvement; 1 point, < 10% involvement; 2 points, 10–25% involvement; 3 points, 25–50% involvement; 4 points, 50–75% involvement, and 5 points, > 75% involvement. Five random fields (200× magnification) per kidney section were used for quantification. The lung injury and intestine injury were assessed using their scoring criteria as previously described [58, 59]. Histological analysis was independently graded by two pathologists.

### Assessment of renal function

Serum creatinine (Scr) and blood urea nitrogen (BUN) were measured with commercially available Creatinine (Cr) Test Kit (C011-2-1, Nanjing Jiancheng Bioengineering Institute, Nanjing, China) and Blood urea nitrogen (BUN) test kit (C013-1-1, Nanjing Jiancheng Bioengineering Institute, Nanjing, China) according to the manufacturer’s instructions.

### MDA, GSH content and non-heme iron assay

Tissue and cellular malondialdehyde (MDA), glutathione (GSH) content, and non-heme iron were measured with commercially available Malondialdehyde (MDA) Test Kit (A003-1, Nanjing Jiancheng Bioengineering Institute, Nanjing, China), Micro Reduced Glutathione (GSH) Assay Kit (A006-2-1, Nanjing Jiancheng Bioengineering Institute, Nanjing, China) and Iron Assay Kit (A039-2-1, Nanjing Jiancheng Bioengineering Institute, Nanjing, China) according to the manufacturer’s instructions.

### Transmission electron microscopy

Renal cortical tissue and cell samples were pre-fixed with 3% glutaraldehyde, fixed with 2% osmic acid (OsO4) and dehydrated with ethanol, and then embedded in epoxy resin. The samples were then stained with uranyl acetate and lead citrate, and ultrathin sections (50–70 nm) were made. The images were obtained under the Hitachi H-7650 transmission electron microscope (H-7650, Hitachi, Tokyo, Japan). Statistical methods of mitochondrial length: three high-power fields (×20,000) were randomly selected from each cell to measure the mitochondrial length. 3–5 cells were randomly selected under each experimental condition, and the total number of mitochondria measured was not less than 60.

### Statistical analysis

Data are expressed as means ± SD. Statistical analyses were performed using Prism 8.3.0 software. Compare between multiple groups with one-way ANOVA followed by Tukey’s post hoc test. Kaplan–Meier survival curves and pairwise log-rank test were used to estimate the survival between groups. Significance was determined when the *P* value was less than 0.05.

## Supplementary Information

**Additional file 1: Fig. S1.** CLP induced AKI. Mice were subjected to CLP, and the kidney samples were collected at the indicated time. (A-B) Shown are representative Hematoxylin-eosin (HE) stains and Periodic Acid-Schiff (PAS) stain. (C) Histological analyses of renal tubular injury. (D) Quantitative analyses of IHC stain of NGAL. (E) Representative immunohistochemistry (IHC) images for NGAL. (F-G) Quantitative analyses of Scr and BUN. n = 6 mice/group, mean ± SD were presented. **P *< 0.01*.

**Additional file 2: Fig. S2.** Specific inhibitor of ferroptosis ameliorates LPS-induced cell death. (A) Viability curves for HK-2 cells treated with different concentrations (0, 0.001, 0.01, 0.1, 1, 10 and 100 ug/ml) of LPS for 24 h. (B) Viability curves for HK-2 cells treated with LPS (1 ug/ml) for different times (0, 1.5, 3, 6, 12, and 24 h). HK-2 cells were pretreated with or without liproxstatin-1(10 µM) or DFO (100 µM) 0.5 h, followed by LPS (1 ug/ml) for 8 h. (C) Fold change of cell viability. (D) Visualizationof cell viability were evaluated by phase-contrast microscopy. n = 3, mean ± SD werepresented. **P *< 0.01*.

**Additional file 3: Fig. S3.** MCTR1 inhibited erastin-induced ferroptosis by Nrf2 signaling. HK-2 cells were transfected with or without Nrf2 siRNA (30 nM) for 48 h and then treated with MCTR1 (100 nM) 0.5 h, followed by erastin (1 µM) for 24 h. (A, C) Fold change of cell viability. (B, D) Visualization of cell viability was evaluated by phase-contrast microscopy. n = 3, mean ± SD were presented. **P *< 0.01*.

## Data Availability

All data generated or analyzed during this study are included in this article.

## Abbreviations

SA-AKI: Sepsis-associated acute kidney injury
Nrf2: Nuclear factor-erythroid-2-related factor 2
MCTR1: Maresin conjugates in tissue regeneration 1
SPMs: Specialized pro-resolving mediators
CKD: Chronic kidney disease
ESRD: End-stage renal disease
ATN: Acute tubular necrosis
DNA: Deoxyribonucleic acid
CNC-bZip: Cap “n” collar family of basic region leucine zipper transcription factors
FTH: Ferritin
FPN: Ferroportin
NQO1: NAD(P)H:quinone oxidoreductase 1
SLC7A11: Solute carrier family 7 member 11
GSS: Glutathione synthase
CLP: Caecal ligation and puncture
AKI: Acute kidney injury
GPX4: Glutathione peroxidase 4
PTGS2: Prostaglandin-endoperoxide synthase 2
GSH: Glutathione
MDA: Malondialdehyde
TECs: Renal tubular epithelial cells
DFO: Deferoxamine
NGAL: Neutrophil gelatinase-associated lipocalin
Scr: Serum creatinine
BUN: Blood urea nitrogen
LPS: Lipopolysaccharide
EC50: Maximal effect
ROS: Reactive oxygen species
GAPDH: Glyceraldehyde-3-phosphate dehydrogenase
CCK-8: Cell counting kit-8
