# Supplementary figures and images for: Maresin conjugates in tissue regeneration-1 suppresses ferroptosis in septic acute kidney injury
Source: Cell Biosci. 2021 Dec 27;11:221. doi: 10.1186/s13578-021-00734-x (PMC8711186; doi:10.1186/s13578-021-00734-x)

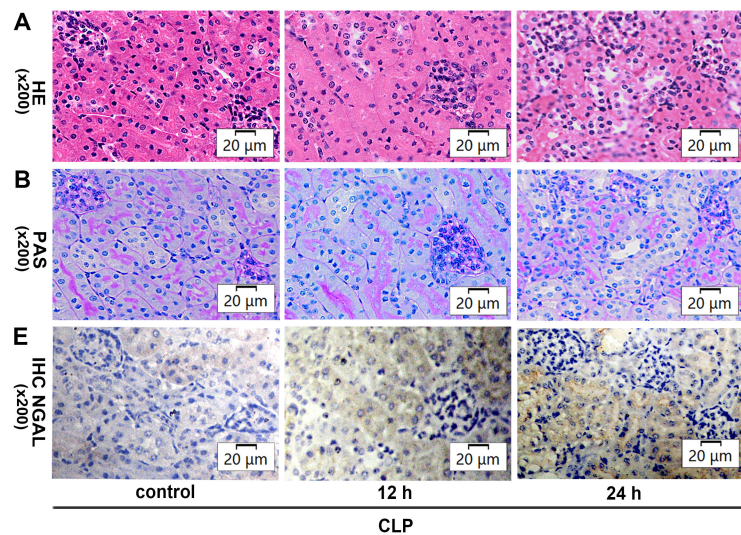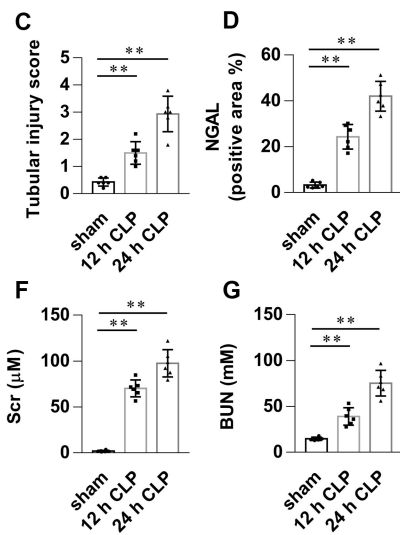

Supplement: Supplementary file 1 — Additional file 1: Fig. S1. CLP induced AKI. Mice were subjected to CLP, and the kidney samples were collected at the indicated time. (A-B) Shown are representative Hematoxylin-eosin (HE) stains and Periodic Acid-Schiff (PAS) stain. (C) Histological analyses of renal tubular injury. (D) Quantitative analyses of IHC stain of NGAL. (E) Representative immunohistochemistry (IHC) images for NGAL. (F-G) Quantitative analyses of Scr and BUN. n = 6 mice/group, mean ± SD were presented. **P < 0.01. [file 13578_2021_734_MOESM1_ESM.pdf]

**A**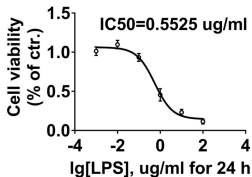**B**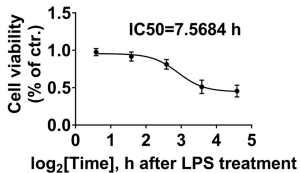**C**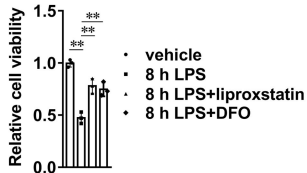**D**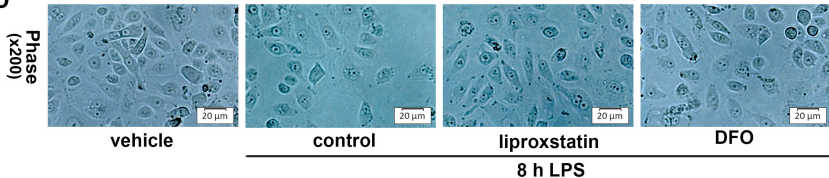

Supplement: Supplementary file 2 — Additional file 2: Fig. S2. Specific inhibitor of ferroptosis ameliorates LPS-induced cell death. (A) Viability curves for HK-2 cells treated with different concentrations (0, 0.001, 0.01, 0.1, 1, 10 and 100 ug/ml) of LPS for 24 h. (B) Viability curves for HK-2 cells treated with LPS (1 ug/ml) for different times (0, 1.5, 3, 6, 12, and 24 h). HK-2 cells were pretreated with or without liproxstatin-1(10 µM) or DFO (100 µM) 0.5 h, followed by LPS (1 ug/ml) for 8 h. (C) Fold change of cell viability. (D) Visualizationof cell viability were evaluated by phase-contrast microscopy. n = 3, mean ± SD werepresented. **P < 0.01. [file 13578_2021_734_MOESM2_ESM.pdf]

**A**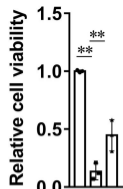

- vehicle
- 24 h erastin
- 24 h erastin+MCTR1

**B**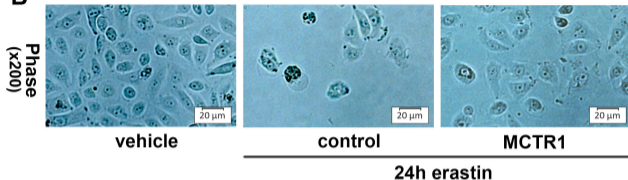**C**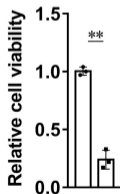

- 24 h erastin+MCTR1+control siRNA
- 24 h erastin+MCTR1+Nrf2 siRNA

**D**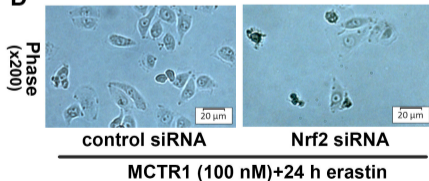

Supplement: Supplementary file 3 — Additional file 3: Fig. S3. MCTR1 inhibited erastin-induced ferroptosis by Nrf2 signaling. HK-2 cells were transfected with or without Nrf2 siRNA (30 nM) for 48 h and then treated with MCTR1 (100 nM) 0.5 h, followed by erastin (1 µM) for 24 h. (A, C) Fold change of cell viability. (B, D) Visualization of cell viability was evaluated by phase-contrast microscopy. n = 3, mean ± SD were presented. **P < 0.01. [file 13578_2021_734_MOESM3_ESM.pdf]
